# Optimization of extrusion conditions for development of high quality rice-lupin-pumpkin based extruded snack food

**DOI:** 10.1016/j.heliyon.2024.e40913

**Published:** 2024-12-04

**Authors:** Yeshambel Dagnaw Alefew, Abebaw Teshome Tiruneh, Tadesse Fenta Yehuala

**Affiliations:** a Department of Food Engineering, Bahir Dar Institute of Technology, Bahir Dar University, Bahir Dar, Ethiopia; b Food and Nutrition Centre, Bahir Dar Institute of Technology, Bahir Dar University, Bahir Dar, Ethiopia

**Keywords:** Blending ratio, Extruded snack, Lupin, Nutritional composition, Physical and functional properties, Optimization, Pumpkin, Rice, Sensory

## Abstract

This study was examined to optimize extrusion conditions (barrel temperature, feed moisture, and blending ratios of rice, lupin, and pumpkin flour) during processing high-quality extruded products using a twin-screw extruder. A three-factor with three-level response surface methodology with a Box-Behnken design, was applied to evaluate the effects of selected processing conditions: blending ratios of lupin (10–20 %), barrel temperature (115–155 °C) and feed moisture content (14–20 %) on the functional, nutritional and sensory characteristics of the produced snack food. The independent variables significantly affected the nutritional, functional, and physical properties of the extruded snack food. A higher proportion of lupin showed higher bulk density but lower expansion ratio and water solubility index of the extrudates while higher feed moisture content led to a reduction in expansion ratio and water-soluble index of the extruded snack food. Lupin substitution and pumpkin flour addition significantly increased the protein, crude fiber, ash, fat, β-carotene, iron, and zinc contents of the white rice-lupin-pumpkin extruded snack, whereas the total carbohydrate content was reduced. The sensory evaluation showed that it was possible to incorporate lupin flour up to a certain level without affecting consumer acceptance. A blending ratio of 15 %, feed moisture of 14 %, and barrel temperature of 155 °C produced the extrudate with a desirability value of 0.75.

## Graphical abstract

**Figure:**
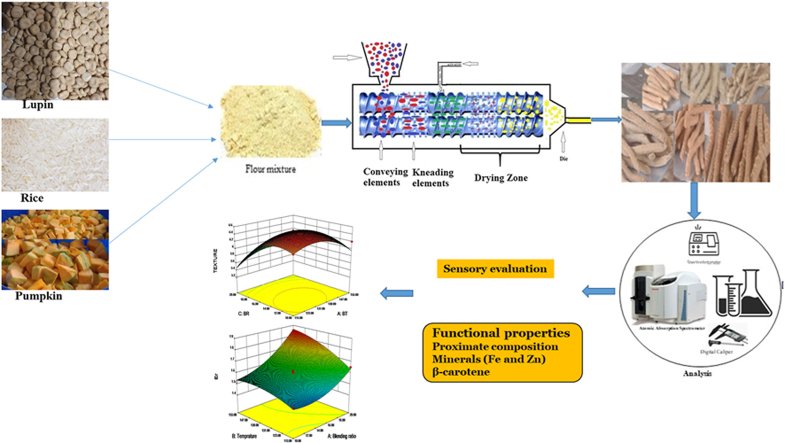

## 1 Introduction

Globally, the snack food industry is growing bigger and more important every day [1]. Extruded snack foods are a subset of ready-to-eat meals that have seen significant expansion in recent years due to reasons including consumer demand (convenience, value, appealing appearance and texture, and suitability for consumption and simplicity of preparation and storage) and availability [[2], [3], [4]]. Snack foods, however, typically contain starch, wheat, sugar, and fat as their main ingredients, making them low in nutritional value and high in calories [3,5]. Since consumers are increasingly demanding better food options, the snack food sector has worked to improve the nutritional content of extruded products by boosting the composition of bioactive phytochemicals [3].

One of the commonly adopted processing techniques by food industries is extrusion, which employs continuous mixing, shearing, forming, texturing, cooking, compressing, and forcing a molten material under high pressure, through a narrow opening (die) to develop a novel food product [6,7]. When the pressure suddenly drops as the material exits the aperture, water turns into steam, which causes the material to expand [6,8]. In addition to the physical changes, the process also gelatinizes starch, denatures proteins, solubilizes fiber, and causes biopolymers to crosslink. This explains why the material's functional and chemical properties underwent such drastic modifications. Extrusion has several advantages over other heat-treating food processing methods, including baking, autoclaving, cooking, and roasting, including little to no nutritional value loss, cheap cost, great productivity, and no process effluents [6,9].

For many customers, snacks play a significant role in their daily calorie and nutritional intake. Starch-based products made from rice, maize, wheat, and potatoes are the most often used raw ingredients for snacks. The nutritional value of cereal-based extruded snack foods can be improved by adding additional components from protein-rich pulses and oilseeds, pigmented maize types, fruit, and vegetables [5,10].

Rice (*Oryza sativa L.*), the second most eaten food crop, is a staple cereal grain for a significant portion of the global population. More than one-fifth of all calories consumed by people globally are delivered through it [11]. White rice has a high carbohydrate content (75–80 %), contains 7–7.77 % protein, 0.23 % fat, 0.41 % ash, and 0.73 % fiber. It also contains vitamin B-complex vitamins like riboflavin, thiamin, and niacin [12,13]. Moreover, white rice flour has become an attractive ingredient in the extrusion industry as a result of its distinctive characteristics such as good-looking white color, bland taste, hypoallergenicity, and simplicity of digestion [14]. It provides affordable, ready-to-eat, gluten-free snacks for around 80 % of food consumption [15]. Because they only contain a modest amount of the essential amino acids, the proteins in cereals are of low biological relevance. They are therefore frequently supplemented with lysine or pulse protein to create wholesome snack items [14].

A valuable and traditional legume food crop, lupin (*Lupinus albus*) can grow in a variety of soil types and climatic conditions. Due to their nutritional and practical qualities, lupin seeds have become more popular as human food [[16], [17], [18]]. Lupin seeds contain a higher protein content, ranging between 33 and 47 %. In addition to having significant levels of dietary fiber, fat, and ash, it also has certain useful qualities, including emulsifying and noodle solubility [19]. In Ethiopia, white lupin (*Lupinus albus*) locally known as “*Gibto*”, is traditionally eaten as roasted snacks, a component of regional soups, and a spice for stews and sauces (called “*Shiro*”) as other typical legumes like peas and other beans [20,21]. Additionally, lupin is used in the preparation of regional alcoholic beverages like *Areke* or *Gibto* *Areke* as well as other food products, particularly in the northwestern part of the country. Lupin seeds are debittered by roasting them and soaking them for 3–7 days in a river or spring of water [22]. While it performs well in the baking industry in many of the nations where the crop is cultivated (the Middle East, central Europe, and Australia). Although lupin is used in diverse traditional foodstuffs, it is an underutilized legume in Ethiopia. Given that lupin is less expensive than other legumes, some researchers have investigated its potential to enhance nutritional profile, textural quality, and recipe cost efficiency [19]. Lupin is a great supplement to cereal proteins that are only lysine-rich because it contains the three key macronutrients (protein, dietary fiber, and carbohydrates) necessary for a balanced diet [18]. Additionally, it was discovered to have a wide variety of phytochemicals, including phytosterols and antioxidants, which may benefit health [19]. Moreover, including lupin proteins in our diet on a regular basis has numerous positive benefits for the management and prevention of several metabolic illnesses like diabetes mellitus and coronary heart disease [23].

Pumpkin (*Cucurbita moschata L.*) is a significant vegetable due to its high nutritional value and advantageous health effects. It is a rich source of carotenoids (α- and β-carotene), which give it its yellow or orange color [24,25], as well as water-soluble vitamins, phenolics, flavonoids, amino acids, carbohydrates, calcium, and phosphorus. It was usually eaten freshly boiled, steamed, or processed as foods like soup or curry [26]. Because of its high nutritional content, bioactive properties, and simplicity of use in subsequent processing into for a variety of products such as breads, crackers, and gluten-free pasta, pumpkin can also be processed into flour, which can serve as an alternative raw material in food preparation. In bakery and confectionary products, pumpkin flour can be utilized as a concentrated source of beta-carotene. This flour could be used due to its sweetness, flavor, deep yellow-orange color, and substantial amount of nutritional fiber [26,27]. In contrast to other forms of flour from cereals, tubers, and roots, its use in food formulations is considerably less [24].

Numerous studies have shown that Ethiopia's rural populations struggle with food insecurity and suffer from chronic protein and energy malnutrition [17]. According to the results of the 2007 census, over 83 % of Ethiopians, the majority of whom are women living in rural areas, experience food insecurity compared to the recommended daily consumption of 2100 kilocalories per person [28]. Malnutrition has an impact on the health and finance status of a rural community [29].

Food diversity, food fortification, and mitigation of loss of the nutritional value of proteins during food processing are essential to combating energy, protein deficiency, insufficient vitamins, and minerals [30]. As a result, this has prompted planning for the development of neglected and underutilized crops as alternatives to current staple crops [31,32].

Therefore, enriching snack foods, including biscuits, bread sticks, cookies, rice pudding, pastries, and crisps as part of a diet-based plan may help to reduce malnutrition. Thus, blending rice flour with lupin and pumpkin flour to produce snacks has the potential to provide macro- and micro-nutrients including protein, pro-vitamin A carotenoids, iron, zinc, and additional bioactive substances (dietary fibers and phenolic compounds) to extrudates [33]. Such blending can offer an extra color to extrudates imparted from pumpkin flour in addition to contributing toward value addition, nutrition, and bioactive components. Additionally, it can lower the cost of production [19]. The experimental design known as response surface methodology (RSM) uses a variety of mathematical and statistical techniques to develop, optimize, and improve a process or product. By mapping a response surface across a specific region of interest, this technique ascertains the link between responses and independent factors. To find out how varied operational conditions affected the end product's quality, RSM was used to create extruded snacks made from various cereals, legumes, and fruit and vegetables [34,35].

In light of this, this study aimed to use a twin-screw extruder to develop and assess nutritious and high-quality extruded snacks using reasonably priced, readily available raw materials. Additionally, a response surface methodology was employed to optimize the process conditions (blending ratio, feed moisture content and barrel temperature) in order to maximize the extrudates' physicochemical and functional properties, and sensory quality from blends of rice-lupin-pumpkin flour.

## 2 Materials and methods

### 2.1 Gathering of experimental materials

Rice (*x-jegna*) was collected at the Woreta Agricultural Research Center in Ethiopia. The rice was carefully cleaned to get rid of immature seeds, poor grains, and extraneous objects. The rice was then milled to remove its husks and bran using a twin blower and fine chaff rice milling equipment (NPF-90, Jiangsu Jinggu Rice Milling Ltd., China). Using a disk attrition miller, white rice samples were ground into flour with a 500 μm mesh screen particle size. The flour was stored at 4 °C in plastic bags prior to being used for extrusion.

### 2.2 Preparation of flour from dried lupin and pumpkin

White lupin and a local species of pumpkin were purchased from the local market in Bahir Dar City, Ethiopia. White lupin was manually cleaned to get rid of superfluous items, bad grains, and immature seeds. The lupin sample (5 kg) was cleaned, rinsed, and immersed in water for 18 h. The seeds from the presoaked sample were then cooked for an hour (1:3 ratio of seeds to water) to make softer the seeds and destroy sensitive to temperature anti-nutritional elements such as trypsin inhibitors. The boiled kernels were left to debitterize for a five-day period at ambient temperature (roughly 25 °C) in neutral-pH city drinking water. Every 4 h, the soaking water was replaced once with fresh water [36]. Following each round of soaking, the complete seeds were manually dehulled, and the seeds were then dried in the sun for 48 h. The dried seeds were milled into 500 μm mesh particles using a disk attrition miller at a cottage grain milling facility in Bahir Dar, Ethiopia. They were then sealed in low density polyethylene bags and kept at 4 °C until extrusion [36].

The pumpkin samples underwent manual peeling, followed by the removal of seeds to isolate the edible part and a thorough rinse under tap water to eliminate any remaining contaminants [37,38]. The French vertical cutter mixer was used to chop the trimmed pumpkin. The chopped pumpkin was dried in a hot air oven (DHG-9140 Drying oven, China) at 65 °C for 16 h [26,38,39] after being exposed to the sun to eliminate the surface moisture. Using an all-purpose batch mill (Ultra Centrifugal Mill ZM 100, Germany), the dried pumpkin was ground and sieved to a 500 mesh particle size. It was then wrapped in a polyethylene bag and stored at room temperature until it was time to extrude.

### 2.3 Formulation of composite flours

Different weight (dry matter basis) ratios of white rice, lupin, and pumpkin flour were mixed in the following ratios: 70:20:10 %, 75:15:10 %, and 80:10:10 %, respectively, with 100 % white rice as a control. In each trial sample, 10 % pumpkin flour was used to improve the nutritional value, color, and flavor. The flour combinations were prepared using a ribbon mixer (Model AB, Alvan Blanch Type, England) and blended for 20 min. Once they were ready to be extruded, they were airtight in bags made from plastic and kept at room temperature.

### 2.4 Experimental design

Processing factors that are white rice-lupin-pumpkin blending ratio, temperature in the barrel and feed moisture levels were varied into three levels (Table 1) to attain a second-order and reliable optimization model (Equation (1)) as explained by Lazic [40]. A combination of the response surface technique and full factorial design (3^3^), In line with Myers et al. [41], was designed to evaluate the impact of each factor's contribution, their interaction, and to enhance the quality of extrudates made using white rice. As responses (dependent variables), it was decided to use physical and functional properties (bulk density, expansion index, water solubility and absorption index), proximate composition, β-carotene, iron, zinc, and sensory characteristics. Response surface analysis and the Box-Behnken Design were used to assess 15 experimental runs. To confirm the applicability of the model, the analysis of variance (ANOVA) generated by “Design-Expert®" Version 8.0.6 (Stat-Ease, Inc., Minneapolis, MN) software was examined for lack of fit, coefficient of determination (R^2^), and Fisher test value (F-value). The statistical significance of the model and model variables was determined at the 5 % probability level (P ≤ 0.05). Each model was expressed in terms of coded factors, regardless of statistically insignificant terms. Factors exhibiting interaction were kept in the models to maintain hierarchy despite their insignificance.

(1) *Y* = b_0_+b_1_B + b_2_BT + b_3_FM + b_11_B^2^+b_22_BT^2^+b_33_FM^2^+b_12_BBT + b_13_BFM + b_23_BTFM+ɛ

Where Y = the response, B = blending ratio (%), BT = barrel temperature (°C), FM = feed moisture (%), b_0_ = intercepts, b_1_, b_2_, b_3_ are linear, b_11_, b_22_, b_33_, = are quadratic and b_12_, b_13_, b_23_, = are interaction regression coefficient terms and *ε* is the random error term, represents the combined effects of all variables not included in the model.

**Table 1:** Experimental design of the study.

No	Processing factors	levels		
		1	2	3
1	Blending Ratio (%)	10	15	20
2	Barrel Temperature (°C)	115	135	155
3	Feed Moisture (%)	14	17	20

To analyse the extruded snack foods chemical, functional, and physical properties in more detail, the optimal processing factors were selected through the application of numerical optimization of multiple responses. Data were presented as the mean ± SD (standard deviation) of triplicate determinations. Tukey pairwise comparisons were carried out by Minitab version 19.2 to identify treatments that differed significantly from one another at p ≤ 0.05 where an ANOVA revealed significant effects (p < 0.05).

### 2.5 Extrusion cooking and process parameters modification

The extrusion process was carried out by adjusting the process conditions in line with the design using a twin-screw extruder (Model: Clextral, BC-21 N0 194, Firminy, France). A Eurotherm temperature controller (Eurotherm Ltd., Worthing, UK) was used to monitor the temperature of each of the extruder's three heating zones on a separate control panel board while the wheat was fed into the machine in alternation. It was necessary to calibrate and adjust the water and flour feed flow rates (via trial and error) prior to the primary extrusion cooking procedure. Then, flour blends were extruded at a feed rate of 150 g/min (9 kg/h) and a screw speed of 150 rpm. The barrel temperature of the extruder at zone three was varied at 115 °C, 135 °C, and 155 °C. These temperature ranges were chosen using physical observation during preliminary extrusion tests, as well as measurements of the expansion ratio and the precise length of the extruded samples. Using the hydration equation [42] and a constant screw speed of 150 rpm, the dough's feed moisture within the barrel was adapted by adding water to the extruder at levels of 14 %, 17 %, and 20 % (chosen in light of the initial extrusion trial outcomes) in the blends.

The extrudates were gathered, labelled, and permitted to cool for half an hour at ambient temperature for the fundamental measurements of length, weight and diameter. This was done once the extrusion process parameters reached steady states as intended. To regulate the size and expansion of the extrudates and increase their shelf life, the extrudates were gathered under steady-state conditions and air dried using a convection air dryer at 85 °C. These samples were then labelled, packed in zipped plastic bags, and kept at room temperature while functional, physicochemical, and sensory studies were conducted [1].

### 2.6 Determination of produced snack food

#### 2.6.1 Expansion ratio (ER)

The expansion ratio was computed using the Digital Caliper model CLEKAHO, CCCP, Russia, with an accuracy of 0.01 mm. This was done by dividing the average cross-sectional diameter of the extruded product by the die's diameter, following the procedures of Fan et al. [43] and IbanogŇlu et al. [44].

#### 2.6.2 Bulk density (BD)

Weight divided by volume was used to determine the extruded snacks' bulk density (g/cm3), assuming that the extrudate had a cylindrical shape [45].

#### 2.6.3 Water absorption index (WAI) and water solubility index (WSI)

Using Anderson's approach [46], the functional properties (WAI and WSI) of extruded snacks were evaluated. A small amount of 2g of extrudates flour was suspended in 25 ml of distilled water and allowed to stand for 30 min before being gently mixed. The mixture was then centrifuged (L-530 Tabletop Low Speed Centrifuge, China) at 3000g for 15 min. The supernatants were transferred into a known-weight evaporating dish and baked for 3 h at 105 °C. The WAI was the ratio of the weight of the gel that remained after the supernatant was removed to the weight of the initial dry solids (Equation (2)). According to Equation (3), the WSI was the weight of dry solids in the supernatant expressed as a percentage of the initial sample weight.

(2) $$\text{WAI}\;\left(g/g\right)=\frac{\text{Weight}\;\text{gain}\;\text{by}\;\text{gel}}{\text{Dry}\;\text{weight}\;\text{of}\;\text{extrudate}}$$

(3) $$\text{WSI}\left(\%\right)=\frac{\text{Weight}\;\text{of}\;\text{dry}\;\text{solid}\;\text{in}\;\text{supernaten}\;t}{\text{Dry}\;\text{weight}\;\text{of}\;\text{extrudate}}\times 100$$

### 2.7 Proximate composition analysis

According to Tiruneh et al. [47], the AOAC (2005) Method numbers 925.10, 923.03, 962.09, 979.09, and 920.39 were used to measure the contents of moisture, ash, crude protein, crude fiber, and fat. The total carbohydrate content (CHO) was detrmined by Monro and Burlingame [48] via the following formula: 100- (% moisture + % fiber + % protein+ % ash and % fat). Based on Regulation (EC) No. 1169/2011 of the European Parliament and of the Council, of October 25, 2011 [49], the gross energy value of the various extruded products was determined using the following equation: E (kcal/100g dw) = 4 × (g protein + g carbohydrate) + 2 × (g fiber) + 9 × (g fat). According to Algarni et al. [50], mineral concentrations (Fe and Zn) of raw materials and extrudates were assessed using an atomic absorption spectrometer (Model: NOVVA AAS 400P, Germany) in compliance with the standard procedures of AOAC (2012).

### 2.8 Determination of β-carotene content

After the material (approximately 1g) was extracted using around 15 ml acetone and a few crystals of anhydrous sodium sulphate, the amount of β-carotene was measured. The supernatant was decanted into a beaker. The procedure was repeated twice before the combined supernatant was moved to a different funnel and thoroughly mixed with 15 mL of petroleum ether. Two layers separated upon standing; the top layer was collected into a 100 ml volumetric flask, and petroleum ether was added to increase the volume to 100 ml [50]. The lower layer was discarded. Using an Agilent Cary 60 UV–Vis Spectrophotometer (USA) set to 452 nm, the absorbance of the extracts was measured using petroleum ether as a blank [51]. The amount of β-carotene was calculated using Equation (4).

(4) $$\beta -carotene\;\left(\frac{mg}{100g}\right)=\frac{Optical\;density\;of\;sample\;x\;13.9\;x\;10^{4}\;x\;100}{Sample\;weight\;\left(dp\right)\;x\;560\;x\;10000}$$

### 2.9 Sensory acceptability of the extrudates

In order to assess the sensory acceptability of the extrudate samples, 25 semi-trained volunteer panelists (15 men and 10 women) with ages ranging from 25 to 35 were selected at random from among postgraduate students and staff at Bahir Dar University's Department of Food Engineering. Panelists were randomly assigned to receive pieces of extrudate (4 cm in length) from each experimental samples (15 samples) in order to rate the likeness of various sensory attributes (color, taste, texture, flavor, and overall acceptability) using a nine-point hedonic scale questionnaire (9 = like extremely, 8 = like very much, 7 = like moderately, 6 = like slightly, 5 = neither like nor dislike, 4 = dislike slightly, 3 = dislike moderately, 2 = dislike very much and 1 = dislike extremely) [14,52,53]. The sensory evaluation was carried out in a laboratory. In between samples, drinking water was available to rinse and clean the mouth. The samples were presented in a random sequence as pieces, 4 cm in length, in three-digit coded dishes. They were served at room temperature (25 °C) in typical lighting conditions. As previously noted, the panelists used a variety of characteristics to compare each extrudate to the others throughout the sensory analysis. The average score was calculated for each of the 25 panelists based on several of attributes [54].

## 3 Results and discussion

### 3.1 Composition of white rice, lupin and pumpkin flour

Table 2 displayed the chemical composition of the flours made from white rice, pumpkin, and lupin. A significant difference (P ≤ 0.05) was found in the proximate composition, iron, zinc, and β-carotene concentration among the three varieties of flour, except for the fat level of pumpkin flour and white rice (Table 2). The moisture percentages of lupin flour (7.8 %), pumpkin flour (8.8 %), and white rice flour (9.4 %), were as follows. As demonstrated by the findings, the moisture content values of flours fall within the permissible range (≤10 %) designed to maintain shelf stability [15]. Due to its superior ability to store water compared to other raw materials, white rice flour had a higher moisture content than lupin and pumpkin flour. In this study, white rice flour's crude protein, crude fiber, ash, fat, total carbohydrate, and energy contents were 7.2 %, 1.1 %, 0.6 %, 1.8 %, 79.8 %, and 359.7 kcal/100g, respectively. As with lupin and pumpkin flour, white rice flour has a high carbohydrate content (79.8 %). Similar proximate composition results to those of Philipp el al. [55] were shown by white rice flour. In this investigation, the lupin flour's crude protein, crude fiber, ash, fat, total carbohydrate, and energy contents were 38.1 %, 8.4 %, 9.9 %, 2.7 %, 33 %, and 380.1 kcal/100g, respectively. For lupin flour, Getachew et al. [34] reported comparable findings. Compared to white rice and pumpkin flour, lupin flour has a higher proportion of protein, crude fiber, fat and energy [50]. Pumpkin flour contained 4.2 %, 1.3 %, 2.9 %, 5.5 %, 77.3 %, and 343.5 kcal/100g of crude protein, crude fiber, ash, fat, total carbohydrate, and energy, respectively. For pumpkin flour, Pongjanta et al. [56] and Dhiman et al. [57] showed comparable findings. The proximate study revealed that lupin flour can contribute to the extrudate produced by partial replacement of white rice flour with lupin flour due to its high protein, crude fiber, fat, and energy content. While pumpkin flour can contribute to the extrudate processing by partial substitution of white rice flour with pumpkin flour due to its high carbohydrate, ash, and crude fiber contents.

**Table 2:** Proximate composition of rice, lupin and pumpkin flour.

Flour	Moisture (%)	Crude protein (%)	Fat (%)	Ash (%)	Crude fiber (%)	CHO (%)	Energy (kcal/100g)	Iron (mg/100g)	Zinc (mg/100g)	β-carotene (mg/100g)
Rice	9.4 ± 0.10^A^	7.2 ± 0.25^B^	1.1 ± 0.15^B^	1.8 ± 0.10^C^	0.6 ± 0.10^C^	79.8 ± 0.15^A^	359.7 ± 0.65^B^	2.6 ± 0.10^C^	1.9 ± 0.20^C^	ND
Lupin	7.8 ± 0.15^C^	38.1 ± 0.32^A^	8.4 ± 0.40^A^	2.7 ± 0.25^B^	9.9 ± 0.40^A^	33.0 ± 0.82^C^	380.1 ± 3.41^A^	8.5 ± 0.01^A^	5.3 ± 0.02^A^	ND
Pumpkin	8.8 ± 0.25^B^	4.2 ± 0.21^C^	1.3 ± 0.21^B^	5.5 ± 0.10^A^	2.9 ± 0.16^B^	77.3 ± 0.23^B^	343.5 ± 0.45^C^	7.2 ± 0.31^B^	2.8 ± 0.15^B^	18.1 ± 0.36

^A,B,C^ The results were presented as means ± standard deviation (n = 3); Means that differ by superscript within a column indicate a significant difference (p < 0.05). CHO = Total carbohydrate and ND denotes “Not Detected”.

The iron, zinc, and beta-carotene concentrations in the white rice, lupin, and pumpkin flour samples varied significantly (P ≤ 0.05). Lupin flour had the highest iron and zinc concentrations (8.5 mg and 5.3 mg, respectively) [57,58], followed by pumpkin (7.2 mg and 2.8 mg, respectively) [59,60] and white rice flour (2.6 mg and 1.9 mg, respectively) [11,50]. The mineral content of lupin in this study is higher than that reported by Ertaş and Bilgiçli [16] and comparable to that of Ka and Chandravanshi [61]. According to Algarni et al. [50], the extent of foreign materials, impurities, varieties, different processing and measuring methods, as well as various growing conditions (such as geographic, seasonal differences in weather, and soil characteristics) could all contribute to the variation in chemical composition.

White rice and lupin flour contained no detectable amounts of β-carotene, while pumpkin flour had 18.1 mg/100g. These results are almost in agreement with those of Dhiman et al. [60] and Khatib and Muhieddine [61]. Similar to this, Algarni et al. [50], Swamy et al. [62], and Wang et al. [63] reported on the β-carotene concentration of white rice flour and lupin flour, respectively. The result indicates that lupin and pumpkin flour can potentially boost the iron and zinc content of extrudates made from white rice. Additionally, using pumpkin flour with white rice flour may raise the β-carotene concentration of extrudates made from white rice.

### 3.2 Impact of extrusion processing parameters and blending ratios on the functional and physical properties extrudate from white rice, pumpkin, and lupin

Table 3 below displays the physical and functional characteristics of the extruded white rice-lupin-pumpkin snack meal. The average proximate composition and micronutrient content of the white rice-lupin-pumpkin extruded snack are shown in (Table 4). Similarly, Table 5 shows the regression coefficients, ANOVA, and R^2^ of the predicted quadratic polynomial models. The f-test and p-value were used to assess the significance of the fitted quadratic model's coefficient.

**Table 3:** Physical and functional properties the rice-lupin-pumpkin based extruded snack.

	Independent Variables			Physical and Functional Properties			
Run	**B**	**BT**	**FM**	ER	BD (g/cm3)	WAI (g/g)	WSI (%)
1	**15**	**135**	**17**	1.6 ± 0.03^BCDE^	0.30 ± 0.10^A^	9.4 ± 0.10^AB^	15.2 ± 0.47^CDE^
2	**10**	**115**	**17**	1.5 ± 0.04^DE^	0.41 ± 0.16^A^	8.8 ± 076^AB^	13.7 ± 0.72^EF^
3	**20**	**135**	**20**	1.8 ± 0.10^BC^	0.54 ± 0.08^A^	9.6 ± 0.15^AB^	16.2 ± 0.06^BCD^
4	**10**	**155**	**17**	1.5 ± 0.08^DE^	0.32 ± 0.13^A^	9.9 ± 0.06^AB^	14.6 ± 0.60^DEF^
5	**15**	**115**	**20**	1.4 ± 0.10^E^	0.50 ± 0.01^A^	8.8 ± 0.15^AB^	14.7 ± 0.21^DEF^
6	**20**	**115**	**17**	1.7 ± 0.05^BCD^	0.42 ± 0.03^A^	7.9 ± 0.12^B^	15.2 ± 0.15^CDE^
7	**15**	**135**	**17**	1.6 ± 0.10^BCDE^	0.30 ± 0.07^A^	9.2 ± 0.26^AB^	15.1 ± 0.23^CDE^
8	**15**	**115**	**14**	1.4 ± 0.08^E^	0.34 ± 0.01^A^	8.6 ± 0.06^AB^	14.5 ± 0.15^DEF^
9	**10**	**135**	**20**	1.5 ± 0.01^DE^	0.39 ± 0.09^A^	10.2 ± 0.61^AB^	13.3 ± 0.93^F^
10	**20**	**135**	**14**	1.7 ± 0.05^BCD^	0.34 ± 0.11^A^	8.2 ± 0.06^B^	15.5 ± 0.48^CD^
11	**15**	**135**	**17**	1.6 ± 0.08^BCDE^	0.31 ± 0.08^A^	9.1 ± 0.32^AB^	14.9 ± 0.44^CDEF^
12	**15**	**155**	**14**	1.6 ± 0.05^BCDE^	0.30 ± 0.10^A^	8.8 ± 0.10^AB^	15.9 ± 0.46^CD^
13	**20**	**155**	**17**	1.9 ± 0.10^B^	0.36 ± 0.06^A^	9.3 ± 0.51^AB^	17.9 ± 0.96^B^
14	**10**	**135**	**14**	1.5 ± 0.13^DE^	0.29 ± 0.05^A^	9.9 ± 0.03^AB^	14.8 ± 0.68^CDEF^
15	**15**	**155**	**20**	1.6 ± 0.08^BCDE^	0.39 ± 0.06^A^	10.3 ± 0.30^A^	16.6 ± 0.98^BC^
	**Control∗**			3.0 ± 0.15^A^	0.30 ± 0.08^A^	9.2 ± 0.30^AB^	50.1 ± 0.40^A^

Notes: ^A,B,C,D,E,F^ The results were presented as means ± standard deviation (n = 3); Means that differ by superscript within a column indicate a significant difference (p ≤ 0.05). B = Lupin Content (%); BT = Barrel Temperature (°C); FM = Feed Moisture (%); ER = Expansion ratio; BD = Bulk density; WAI = Water absorption index; WSI = Water solubility index and control∗ was from 100 % rice.

**Table 4:** Proximate composition and micronutrient content of the rice-lupin-pumpkin based extruded snacks.

Run	Independent variables			Proximate composition of the extruded snack foods							Micronutrients of the extruded products		
	B	BT	FM	Moisture (%)	Protein (%)	Fat (%)	Fiber (%)	Ash (%)	CHO (%)	Energy (kcal/100g)	Fe (mg/100g)	Zn (mg/100g)	β-carotene (mg/100g)
1	**15**	**135**	**17**	6.5 ± 0.92^BC^	12.1 ± 0.36^CDE^	1.7 ± 0.07^ABC^	1.6 ± 0.08^BCD^	1.5 ± 0.11^BCD^	76.6 ± 0.78^BCD^	373.3 ± 4.95^AB^	3.1 ± 0.45^AB^	1.6 ± 0.08^BCD^	3.9 ± 0.52^ABC^
2	**10**	**115**	**17**	6.5 ± 0.76^BC^	12.0 ± 0.50^CDE^	1.5 ± 0.08^BCD^	1.3 ± 0.07^F^	1.4 ± 0.11^BCDE^	77.4 ± 1.04^BC^	373.2 ± 3.62^AB^	2.9 ± 0.40^AB^	1.5 ± 0.12^BCD^	5.0 ± 0.50^A^
3	**20**	**135**	**20**	7.7 ± 0.51^AB^	16.7 ± 0.82^A^	1.7 ± 0.11^ABC^	2.0 ± 0.08^A^	1.6 ± 0.06^ABC^	70.3 ± 1.15^GH^	366.0 ± 1.24^AB^	3.6 ± 0.15^A^	1.8 ± 0.10^AB^	4.6 ± 0.17^AB^
4	**10**	**155**	**17**	6.4 ± 0.47^BC^	11.3 ± 0.44^DE^	1.4 ± 0.14^CD^	1.3 ± 0.13^F^	1.2 ± 0.04^E^	78.4 ± 1.00^B^	373.8 ± 0.14^AB^	2.8 ± 0.17^AB^	1.5 ± 0.09^BCD^	3.2 ± 0.53^BCD^
5	**15**	**115**	**20**	8.0 ± 0.45 ^A^	18.5 ± 0.50^A^	1.6 ± 0.04^BCD^	1.6 ± 0.05^BCD^	1.5 ± 0.11^BCD^	69.1 ± 0.98^H^	365.9 ± 1.65^B^	3.6 ± 0.35^A^	1.5 ± 0.14^BCD^	5.2 ± 0.47^A^
6	**20**	**115**	**17**	7.5 ± 0.76^AB^	13.7 ± 0.64^BC^	2.1 ± 0.18^A^	1.5 ± 0.03^DEF^	1.6 ± 0.09^BCD^	73.7 ± 1.37^EF^	371.3 ± 2.29^AB^	3.7 ± 0.29^A^	1.7 ± 0.10^ABC^	5.1 ± 0.60^A^
7	**15**	**135**	**17**	6.5 ± 0.61^BC^	13.8 ± 0.72^BC^	1.6 ± 0.07^BCD^	1.4 ± 0.13^EF^	1.5 ± 0.08^BCD^	75.2 ± 0.06^CDE^	373.1 ± 3.11^AB^	3.0 ± 0.75^AB^	1.6 ± 0.09^BCD^	4.2 ± 0.55^ABC^
8	**15**	**115**	**14**	6.2 ± 0.53^BC^	14.2 ± 0.72^B^	1.5 ± 0.08^BCD^	1.4 ± 0.01^EF^	1.6 ± 0.07^ABC^	75.1 ± 0.32^CDE^	373.8 ± 2.91^AB^	3.4 ± 0.76^AB^	1.6 ± 0.15^BCD^	5.2 ± 0.35^A^
9	**10**	**135**	**20**	7.4 ± 0.35^AB^	11.2 ± 0.92^E^	1.3 ± 0.15^CD^	1.5 ± 0.07^DEF^	1.4 ± 0.04^BCDE^	77.3 ± 1.10^BC^	368.6 ± 0.78^AB^	2.5 ± 0.12^AB^	1.4 ± 0.09^CD^	3.4 ± 0.40^BCD^
10	**20**	**135**	**14**	6.5 ± 0.50^BC^	13.1 ± 0.90^BCDE^	1.8 ± 0.14^ABC^	1.8 ± 0.09^AB^	1.7 ± 0.08^AB^	75.1 ± 1.44^CDE^	372.7 ± 1.06^AB^	3.5 ± 0.46^A^	1.6 ± 0.13^BCD^	3.8 ± 0.44^ABC^
11	**15**	**135**	**17**	6.4 ± 0.51^BC^	13.4 ± 0.69^BC^	1.6 ± 0.11^BCD^	1.5 ± 0.04^DEF^	1.4 ± 0.13^BCDE^	75.6 ± 0.28^CDE^	373.7 ± 1.86^AB^	3.1 ± 0.40^AB^	1.5 ± 0.09^BCD^	4.0 ± 0.68^ABC^
12	**15**	**155**	**14**	6.2 ± 0.26^BC^	17.3 ± 0.64^A^	1.6 ± 0.09^BCD^	1.6 ± 0.13^BCD^	1.6 ± 0.10^ABC^	71.8 ± 0.49^FG^	373.4 ± 0.32^AB^	3.4 ± 0.16^AB^	1.5 ± 0.08^BCD^	2.2 ± 0.35^D^
13	**20**	**155**	**17**	6.9 ± 0.24^ABC^	13.3 ± 0.70^BCD^	1.8 ± 0.25^AB^	1.8 ± 0.10^ABC^	1.8 ± 0.08^A^	74.3 ± 0.85^DE^	370.6 ± 1.15^AB^	3.6 ± 0.40^A^	2.0 ± 0.13^A^	2.8 ± 0.75^CD^
14	**10**	**135**	**14**	5.7 ± 0.23^C^	12.3 ± 0.61^BCDE^	1.3 ± 0.07^CD^	1.5 ± 0.11^DEF^	1.3 ± 0.13^DE^	77.8 ± 0.31^BC^	375.4 ± 1.64^A^	2.7 ± 0.35^AB^	1.4 ± 0.10^CD^	3.8 ± 0.76^ABC^
15	**15**	**155**	**20**	6.7 ± 0.12^ABC^	14.2 ± 0.76^B^	1.5 ± 0.04^BCD^	1.6 ± 0.10^BCD^	1.6 ± 0.06^ABC^	74.4 ± 0.66^DE^	371.0 ± 0.23^AB^	3.4 ± 0.47^AB^	1.6 ± 0.11^ABC^	3.8 ± 0.44^ABC^
	**Control∗**	7.4 ± 0.10^AB^	6.8 ± 0.36^F^	1.1 ± 0.15^D^	0.6 ± 0.10^G^	1.5 ± 0.06^BCD^	82.5 ± 0.32^A^	368.7 ± 0.68^AB^	2.2 ± 0.10^B^	1.3 ± 0.15^D^	ND		

Notes: ^A,B,C,D,E,F,H^ The results were presented as means ± standard deviation (n = 3); Means that differ by superscript within a column indicate a significant difference (p ≤ 0.05). B = Lupin Content (%); BT = Barrel Temperature (°C); FM = Feed Moisture (%); CHO = Total carbohydrate; control∗ was from 100 % rice and ND denotes “Not Detected”.

**Table 5:** ANOVA, R^2^ and Regression coefficients of quadratic polynomial models for the studied responses of expanded snack models.

Source	ER	BD (g/cm3)	WAI (g/g)	WSI (%)	Moisture (%)	Protein (%)	Fat (%)	Fiber (%)	Ash (%)	CHO (%)	Energy (kcal/100g)	Fe (mg/100g)	Zn (mg/100g)	β-carotene (mg/100g)
Model	0.002	0.005	0.001	0.003	0.002	0.005	0.004	0.004	0.003	0.001	0.004	0.000	0.017	0.008
Linear	0.000	0.001	0.000	0.000	0.000	0.016	0.001	0.001	0.001	0.001	0.001	0.000	0.004	0.001
B	0.000	0.016	0.000	0.000	0.004	0.004	0.000	0.000	0.000	0.000	0.012	0.000	0.001	0.409
BT	0.002	0.007	0.159	0.001	0.013	0.293	0.098	0.017	0.098	0.083	0.130	0.102	0.162	0.000
FM	0.633	0.001	0.449	0.979	0.000	0.127	0.290	0.299	0.447	0.004	0.000	0.934	0.126	0.091
Quadratic	0.009	0.022	0.008	0.289	0.176	0.004	0.042	0.020	0.087	0.002	0.043	0.001	0.166	0.880
B^2^	0.011	0.024	0.139	0.624	0.095	0.008	0.535	0.182	0.619	0.009	0.233	0.049	0.150	0.582
BT^2^	0.067	0.051	0.007	0.090	0.212	0.033	0.433	0.042	0.083	0.013	0.308	0.000	0.170	0.645
FM^2^	0.014	0.018	0.008	0.852	0.193	0.005	0.011	0.012	0.044	0.002	0.011	0.024	0.224	0.889
Interaction	0.199	0.199	0.080	0.042	0.044	0.010	0.414	0.111	0.012	0.003	0.098	0.103	0.082	0.109
B∗BT	0.048	0.461	0.120	0.046	0.229	0.861	0.200	0.101	0.003	0.820	0.472	0.815	0.080	0.507
B∗FM	0.946	0.102	0.032	0.023	0.318	0.022	0.493	0.116	0.376	0.017	0.570	0.032	0.088	0.118
BT∗FM	0.888	0.203	0.660	0.523	0.013	0.004	0.432	0.174	0.115	0.001	0.025	0.234	0.146	0.055
Lack-of-Fit	0.216	0.042	0.464	0.111	0.061	0.753	0.366	0.714	0.510	0.702	0.082	0.188	0.609	0.169
R^2^	97.49	96.09	98.41	96.93	97.15	96.00	96.38	96.52	97.07	98.14	96.37	98.86	93.51	95.20

Where: B = Lupin Content (%); BT = Barrel Temperature (°C); FM = Feed Moisture (%); ER = Expansion ratio; BD = Bulk density; WAI = Water absorption index; WSI = Water solubility index and CHO = Total carbohydrate; Significant at p ≤ 0.05

#### 3.2.1 Expansion ratio

The expansion characteristic of extruded snacks is significant, especially for the acceptability by the consumer. The product's physical dimensions and shape are significantly determined by the ER, which also has an impact on the biting quality and aesthetic appeal. Expansion is a function of viscosity and elasticity of dough governed by ratio of starch, protein and fiber [33]. In this investigation, the extruded snack products' expansion ratios ranged from 1.4 (runs 5 and 8) to 3.0 (control sample) (Table 3). At 155 °C, the expansion ratio was discovered to be greater. It was shown that the level of lupin in the feed material increased as expansion decreased. Maximum expansion was observed with control extrudates (3.0), while reduced expansion was observed with products prepared with 15 % lupin and 115 °C barrel temperature. Both blending ratio and barrel temperature had notable effects (p = 0.000, 0.002) in both linear and quadratic models on the ER of the product, whereas there was no discernible effect by the feed moisture content (Table 5).

While the interaction among the barrel temperature and blending ratio was significant with ER the three independent variables interaction effect (mixing ratio, feed moisture content and barrel temperature) was not significant with ER. The blending ratio (lupin content) was the main significant factor affecting ER. Less expansion was seen in the extruded products with higher lupin concentrations. This fact can be explained by the fact that adding lupin to samples of white rice used to make the extrudates increased the protein content and decreased the starch content [33,50,64]. Starch is a key ingredient that causes dough to expand inside the barrel of extruder, which results in a rise at the die leaving. Further, the premature rupture of air cells or the inhibition of starch matrix puffing throughout the extrusion process may have been caused by the high fiber content of the lupin and pumpkin flour, which may have resulted in a decrease in the size and quantity of internal air cells [5]. Because rice starch is easily expanded, Kumar et al. [14] and Oliveira et al. [64] showed that when the quantity of rice starch increased, so did the ER. Additionally, the bulk of lupin proteins are legumin-like and do not function as proteins; they may therefore prevent full starch gelatinization throughout the extrusion process. According to Devi et al.'s study [65], starch gelatinization was necessary for the organization of extrusion-expanded products, and ER decreased as the composites' soybean percentage increased.

With certain exceptions, it seemed that ER increased as barrel temperature increased. The degree to which water in the extruder is superheated will increase as the temperature rises, which will cause bubbles to form and expand to a slightly greater extent. Numerous researchers have observed similar findings [33,55,66]. Philipp el al. [55] also showed that the extrusion temperature had a comparable impact on the expansion ratio. Moisture content is important for expansion; as feed moisture levels rise, different values of ER extrudates result [67]. Increased feed moisture results in less specific mechanical energy input, which reduces the starch's physicochemical transformation. This causes the growth to be lessened [33,67]. The molecular structure of the amylopectin in the feedstock would change during extrusion, reducing the melt density and reducing the expansion of the extrudate. Foods with higher moisture content would have a smaller pressure differential due to their higher viscosity compared to foods with lower moisture content, which would result in a less expanded product [66]. These findings concur with the research published by Hagenimana et al. [68].

#### 3.2.2 Bulk density

Bulk density, which is directly related to texture, measures how compact the extrudate is. The extrudate's bulk density is also essential for producing larger food items, figuring out whether they will float or sink in water, and calculating how much packaging is needed [50]. According to Table 3, the bulk density (BD) of the study's extruded product ranged from 0.29 g/cm^3^ (run 14) to 0.54 g/cm^3^ (run 3). According to an ANOVA study, bulk density was significantly affected by linear (p = 0.001) interactions between the blending ratio and the barrel temperature, as well as quadratic terms (p = 0.024, 0.018) of blending ratio and moisture content. The three independent variables' interaction effect was not statistically significant (p = 0.199) with BD. Bulk density was directly impacted by the extrusion temperature and the moisture content of the raw material [69].

According to the current study, an increase in lupin flour levels can be linked to an increase in BD. Lupin addition may also boost the amount of protein and fiber in the mixture. Similar to this, adding pumpkin flour could boost the amount of fiber. The highest concentrations of fiber and protein may have an effect on the starch gelatinization process and the rheological properties of the raw materials used in extrusion processes [33,50]. Additionally, this increased BD might be explained by the various crop kinds, blending ratios, and uses of white rice flour [70].

The extrudate density value increases quickly with increased feed moisture at all temperature levels [55]. When the moisture level of the product is high, its BD is also high. This is because the extrudates retain moisture due to insufficient extrusion heating temperatures, which evaporate the moisture [33]. Furthermore, Hagenimana et al. [68] observed that during the extrusion of rice flour, the BD rose as the moisture ratio increased. An increased feed moisture content during extrusion can further reduce the dough's elasticity by plasticizing the melt, which lowers specific mechanical energy and decreases gelatinization, which in turn reduces expansion and raises extrudate density.

On the other hand, it can be shown that BD showed a propensity to grow when moisture content was raised at a high barrel temperature (>115 °C). This might be explained as follows: as the moisture content rose, the viscoelastic characteristics changed, and it's possible that air bubbles compressed inside the extrudates, reducing expansion and increasing BD [10]. While it was not significant in quadratic terms, temperature had a highly significant impact on the density of extrudates made from all types of feed materials [71]. Increased thermal input from a high temperature results in total gelatinization. Low density of products is also caused by structural degradation of protein and starch in high shear environments [72]. The study stating that extrudate viscosity decreased with higher temperature and that lower viscosity effect would enhance the growth of bubbles during extrusion and consequently thereby decreasing the density [55,72] indicated that an increase in the barrel temperature decreased the melt viscosity. When the product comes out of the die from the extrusion process, the heat generated causes moisture to evaporate quickly, leading to an enlarged structure with big alveoli and a lower BD. Cell organization crumples as a result of this action, which often disappears after the product is cooled [73].

It is evident that bulk density decreased when barrel temperature was increased at low moisture content this phenomenon could be the consequence of greater barrel temperature induced starch gelatinization which increases the amount of water trapped inside the extrudates and causes them to expand more resulting in a lower BD. The moisture level of the extrudates in snack foods has a significant impact on the matrix characteristics during the expansion process and serves as the catalyst for the creation of air bubbles. The viscoelastic characteristics of the extrudates may have improved as the barrel temperature rose, producing enough vapor pressure to expand the extrudates as intended. This would have resulted in the creation of a significant number of tiny air bubbles, which would have increased the pressure and ruptured the cell walls, allowing water vapor to escape, extrudates to grow, and BD to diminish [10]. Similar research showing that the BD is inversely related to the degree of expansion was conducted using products made from a blend of pea and rice grits [10].

#### 3.2.3 Water absorption index

A common tool for assessing the level of starch gelatinization is the WAI. It evaluates the consistency of the starch in the water as well as the amount of the swelled starch in the additional water [74]. The extrudates of white rice, lupin, and pumpkin showed a water absorption index ranging from 7.9 g/g (run 6) to 10.3 g/g (run 15) (Table 3). The linear and quadratic models with the different WAI components were significant (p = 0.000, 0.008), according to an ANOVA study. On the other hand, there was little interaction between barrel temperature and WAI, feed moisture content, and blending ratio. The three independent variables (barrel temperature, feed moisture content, and blending ratio) had a substantial interaction effect with WAI (Table 5). When the lupin ratio was increased from 10 % to 20 %, WAI slightly decreased. The rise in lupin has no appreciable impact on the reduction in WAI. This could be because lupin flour has a low concentration of starch that is readily available; decreasing the starch level by adding lupin could affect how much starch gelatinizes in the barrel and lower the product's ability to absorb water. Similar to this, adding sweet lupin or pea-based grits to rice caused to notice a drop in WAI in the extruded product Algarni et al. [50] and Singh et al. [71]. It was discovered that WAI was increased when the temperature was elevated from 115 to 155 °C. The starch is disturbed and has the ability to bind more water to itself as the temperature rises, increasing WAI [11,55].

According to Delgado-Nieblas et al. [10] and Singh et al. [71], WAI also rose in the majority of runs as feed moisture content increased from 14 to 20 %. Because of the decreased viscosity of the feed due to the increased moisture level, starch molecules are able to move freely, which improves heat diffusion and causes more starch to gelatinize. During extrusion cooking, water has a plasticizing effect, resulting in less friction between screws and the starch molecules and the inner barrel wall. This phenomenon slows down the decomposition of starch granules, increasing the WAI of the finished product and increasing its capacity to absorb water [10,55]. This behavior might be caused by the gelatinization phenomenon, which is encouraged by the product's higher moisture content, and the starch being damaged by the higher barrel temperature. The hydrophilic groups of the extruded snacks, primarily starch and soluble dietary fiber, may have been exposed to more light as a result, allowing enhanced water penetration into the pellet structure. The combination of water and heat with the material causes starch gelatinization, which transforms starch molecules into a digestible substance in the extrusion process. The starch molecules are attached to the water that has been absorbed, changing the structure of the starch granules [10,55].

The protein denaturation, starch gelatinization, and crude fiber swelling that occurred during extrusion may be the cause of the higher WAI of extrudates [71]. A high WAI indicates good starch digestibility in vitro because it indicates the degree of gelatinization and dextrinization. WAI is determined by the presence of hydrophilic groups that bind water molecules in the extruded products, mainly starch and soluble dietary fiber [69].

#### 3.2.4 Water solubility index

The water solubility index, which measures how well the extrudate dissolves in water, offers information about the degree of starch gelatinization and protein denaturation that occurs during extrusion [10]. According to this study's extrudates, the WSI ranged from 13.3 % (run 9) to 50.1 % (control sample) (Table 3). The linear model with the various WSI components was statistically significant (p = 0.000), as per the findings of the ANOVA analysis results of (Table 5). With regard to the three independent variables, the interaction effect was significant for the WSI but not for barrel temperature or moisture content. According to statistical research, the linear term was considerably impacted by the barrel temperature and the mixing ratio. As demonstrated by Pardhi et al. [72] and Singh et al. [71], the WSI of extrudates rises with decreasing feed moisture and rising temperature. The addition of pumpkin and lupin flour reduced the WSI of the extrudates. The WSI of extrudates was considerably impacted by the incorporation of lupin flour in the feed mixture. Lupin addition has a big impact on extrudates' WSI when combined with moisture and temperature. The composition of the feed has changed as a result of the incorporation of lupin and pumpkin flour, which has caused changes in WSI. Fiber, carbohydrate, and protein contents are said to have an impact on WSI, according to Algarni et al. [50]. The levels of fiber, protein, and fat in lupin are comparatively higher. High fiber molecule concentrations interfere with the extruded product's continuous structure and prevent elastic distortion throughout the extrusion process [50]. There is an increased WSI found in extruded products because their minor molecular weight components can readily separate from one another under more demanding processing conditions [33].

It is evident that when the barrel temperature was elevated, the WSI values increased consistently for all ranges of feed moisture content. Shearing pressures, high pressure, and temperature all affect the depolymerization of the starch chain, which raises the WSI value of the extrudates [10]. The WSI is directly correlated with the barrel temperature of extruded products, as per the findings of Philipp et al. [55] and Delgado-Nieblas et al. [10]. The quantity of starch gelatinization that might increase the amount of soluble starch and lead to a rise in WSI would increase with rising temperature [55,72]. Low moisture content combined with high barrel temperatures resulted in the greatest WSI values (>14.5 %). This behavior could be explained by the fact that as the barrel temperature rose, the starch and pectin molecules in pumpkin flours began to degrade more quickly, releasing low-molecular-weight chemicals and increasing their solubility in water. Low moisture level could have increased internal friction, leading to severe mechanical damage and an elevated WSI. A lower WSI is the result of lesser leaching of soluble particles due to higher moisture limitations on protein denaturation [55]. WSI dropped as feed moisture content rose, which may have helped reduce friction by reducing WSI. Water functions as a lubricant, which lowers WSI levels. Rice and fish flour co-extrudates [73] and rice-based extrudates [55] have all been observed to have similar effects.

### 3.3 Effect of extrusion processing settings and the blend ratio on the proximate composition and micronutrient content of the extruded snack made from white rice, lupin, and pumpkin

The average results for the proximate composition and micro-nutrient content of extrudates are displayed in Table 4. As expected, adding pumpkin and lupin flour boosted the nutritional value of the extrudates. Because pumpkin flour has high amounts of these components, adding 10 % of it increased the amount of fiber, ash, zinc, iron, and β-carotene significantly (see Table 2). Moreover, as lupin levels rose, so did the values of protein, fat, fiber, and ash. The amounts of moisture and carbohydrates dropped. Due to lupin's high levels of protein, fat, fiber, and ash, these results may be explained. On rice-lupin extrudates, Algarni et al. [50] and Oliveira et al. [64] found a similar observation. The white rice, lupin, and pumpkin snack's moisture content ranged from 5.7 % (run 14) to 8.0 % (run 5). This index is thought to be one of the key factors affecting food products' capacity to remain fresh on the shelf. The low moisture content found in the by-products' is a good thing because moisture levels under 14 g/100 g can inhibit microbiological growth, enhance chemical and enzymatic stability, and lengthen the shelf life of the products [75]. The ANOVA demonstrated a substantial (p = 0.000) linear dependence between the various factors. In the quadratic terms, however, it had no discernible impact (Table 5). With increasing amounts of lupin and moisture at all levels investigated, the white rice-lupin-pumpkin snack's moisture content rose. Higher moisture content in produced snacks may be caused by damaged starch and added beans. These findings concurred with those of Adem et al. [70]. The present study's results supported past investigations that demonstrated rise in feed moisture raised the extruded product's moisture content. The findings of this study indicated that the moisture content of the ready-to-eat snack formed from rice and cowpea flour was slightly higher than the 5.99 % reported by Okunola et al. [76].

The extruded snacks had protein levels ranging from 6.8 % (control sample) to 18.5 % (run 5). The amounts of lupin content had a substantial (p = 0.004) impact on the product's protein content. Based on the ANOVA study, the blending ratio (lupin content) significantly improved the protein content in both linear and quadratic terms (Table 5). Increased lupin flour concentration (10–20 %) had a significant impact on the proximate characteristics of extrudates made from white rice flour. Legume flour added to cereal-based formulations has been shown to improve the balance of their essential amino acids [70]. Given that the addition of high protein, high lysine raw materials is recognized to improve the quality of protein in cereal diets, legumes should complement cereal proteins in terms of their sulfur and lysine content [64]. The findings of the present study were almost similar to those of Devi et al. [65], who reported 12.6 % crude protein from an extruded sorghum-corn-lentil blend. In contrast, Algarni et al. [50] found that rice, sweet potato, and sweet lupin flour mixes contained somewhat less protein (8.60–11.10 %). This would account for their lower readings, given that there was only 10 % lupin in the mixture. According to Ziena and Ziena [77], a mixture of broken rice grains and legumes contained 12.7–14.4 % crude protein. However, the result of the present study is seen to be higher than the stated value (9.44–14.72 %) of Bassinello et al. [78] investigation on gluten-free extrudates made from composite flours of rice and bean.

The extruded snacks used in this investigation had fat contents that ranged from 1.1 % (control sample) to 2.1 % (run 6) (Table 4). According to the ANOVA study, the blending ratio is the factor that has the greatest impact on the final product's fat content (p = 0.000) (Table 5). These results suggest that adding 10–20 % lupin to a cereal system will significantly increase the system's overall fat content. In the quadratic terms, feed moisture content also significantly impacted fat content. This study's findings exceed those of Algarni et al. [50], who indicated that blended rice, sweet potato, and sweet lupin flours contained (0.37–0.57 %) crude fat in extruded snack foods. Similar findings were studied by Pansawat et al. [73], who found that rice flour and fish co-extrudates included 0.19–1.30 % crude fat. The results of this study have a lower fat content (4.0–4.44 %) than the snack meal prepared from rice, Kersting's groundnut, and lemon pomace mixture flours by extrusion technology, as described by Awolu et al. [53]. In contrast to this study, a snack made from rice and soybean by products was reported to have a higher (4.08 %) fat content by Coutinho et al. [75]. This may be because different crop varieties were used, different processing techniques were used while making the flour, and different percentages of each ingredient were used.

As given in Table 4, the prepared snack food's crude fiber content ranged from 0.6 % (control sample) to 2.0 % (run 3). These values indicated a rise in comparison to the control (0.6 %). These variations were related to the pumpkin flour and lupin mixing ratios. The findings indicated that adding lupin and pumpkin flour could provide high fiber snacks with good nutritional qualities [71,79]. White rice-lupin-pumpkin snack's crude fiber was considerably (p = 0.000, 0.017) impacted by blending ratios and barrel temperatures according to an ANOVA study (Table 5). Additionally, BT and FM have quadratic significance. Typically, fibre particles prevent the product from expanding by breaking down the cell walls before the size of the gas bubbles reaches its maximum. Consequently, high fibre content extruded products usually have an unfavorable, compact, harsh, and non-crisp texture [5]. The extruded product used in this investigation had a fiber content that was comparable to that reportedby Algarni et al. [50] and Coutinho et al. [75], which found that a blend of snack food made from broken rice, sweet potatoes, and sweet lupin as well as by-products of rice and soybean had a fiber content ranging from 1.50 to 2.39 %. The outcomes of this investigation were published in processed, ready-to-eat extruded cereals and snack foods that contain flours derived from maize, wheat, and whole wheat. However, they ranged from 2.52 to 3.78 %, which was less than Awolu and Osigwe's [15] findings. The crude fiber content of the finished snacks was unaffected by interactions between moisture contents.

In most of the runs, the amount of lupin flour in the blends rose with the ash concentration. The largest level of ash was found in snack items containing 20 % lupin (Table 4). Extrudates showed ash amounts ranging from 1.2 % (run 4) to 1.8 % (run 13). The lupin content that was utilized in the extrudates manufactured with pumpkin and lupin flour was directly connected with the increased ash content that was observed. Table 5 shows that there was a significant (p = 0.000) linear effect of the amount of lupin flour on the extruded product's ash content. The moisture content and barrel temperature had no discernible effects on the ash content of the snacks that were made. Awolu et al. [53] revealed similar results (1.25–1.81 %) in ready-to-eat extruded snack made from rice flour enriched with kersting's groundnut and lemon pomace flours.

The white rice-lupin-pumpkin blended extrudates' carbohydrate content ranged from 69.1 % (run 5) to 82.5 % (control sample), as shown in Table 4. The snack food made from the control sample was found to have a high carbohydrate content. The smallest carbohydrate content was showed in the mixed ratio of 70 % rice and 20 % lupin. Increased lupin flour concentration in the mixture significantly decreased the extruded product's carbohydrate content. The carbohydrate value of extrudates may also be slightly reduced by the use of pumpkin flour. This resulted from lupin having less carbohydrate content than rice [50]. The feed moisture content and blending ratio had a substantial impact on the extrudates' carbohydrate content in both linear and all quadratic terms (p = 0.000, 0.004). The overall amount of carbohydrate in the produced snacks was unaffected by barrel temperature (Table 5). Similar findings were reported by Coutinho et al. [75], who found that the extruded product made from rice and soybean snack flour contained 79.35 % carbohydrate. The present study presented lower results than those reported by Algarni et al. [50], who reported on 85.02–90.36 % of processed extruded snack food made from broken rice, sweet potatoes, and sweet lupin.

The energy value of the extruded foods used in the current study ranged from 365.9 kcal/100 g (run 5) to 375.4 kcal/100 g (run 14), as indicated in Table 3. According to the ANOVA study, moisture content and blending ratio are the most important (p = 0.000, 0.012) variables influencing the final product's energy content in linear terms, as well as the feed moisture content used in the quadratic model (Table 5). Energy value of the extrudates increased as the quantity of lupin flour in the mixture increased. This resulted from lupin's increased lipid content [75]. The findings of this investigation were less than those of Algarni et al. [50], which were described as 389.79–393.85 kcal/100g in processed extruded snack food from broken rice, sweet potato, and sweet lupin.

The presence of lupin and pumpkin in extruded snack results in an increase in iron and zinc level, as shown in Table (4). The higher mineral content of lupin and pumpkin flours when compared to white rice flour may be the cause of this rise. The iron level showed rising trend and varied from 2.2 mg/100 g (control sample) to 3.7 mg/100 g (run 6). The highest amount was seen in snack foods that included 20 % lupin. Increased iron concentration was evident in the extrudates made using pumpkin and lupin flour, and this increased iron content was directly correlated with the content of lupin used. The extruded snack's iron content was significantly (p = 0.000) impacted linearly by the amount of lupin flour (Table 5). The temperature and moisture content of the barrel had no discernible effects on the iron content of the prepared extruded snacks. Awolu and Osigwe [15] obtained similar results (2.82–3.47 mg/100g) in an extruded snack prepared from rice, kersting's peanut, and lemon pomace flours. The current study's results were higher than those of Algarni et al. [50], and they were reported as 1.67–2.17 mg/100g in processed extruded snack food made from broken rice, sweet potato, and sweet lupin.

The prepared snack products' zinc concentrations ranged from 1.3 mg/100 g (control sample) to 2.0 mg/100 g (run 13). These numbers increased compared to the control (1.3 %). These variations were related to the use of white rice flour in the lupin blending ratios. The findings indicated that adding lupin flour could provide high zinc snacks with good nutritional qualities [50]. The zinc content of white rice-lupin-pumpkin snack products was significantly (p = 0.001) impacted by blending ratios in an ANOVA analysis (Table 5). Algarni et al. (2019) [50] reported similar findings in a ready-to-eat extruded snack made from a composite flour containing rice, sweet potato, and sweet lupin flours.

White rice-lupin-pumpkin extruded snacks' β-carotene contents ranged from not found (control sample) to 5.2 mg/100g (runs 5&8) (Table 4). β-carotene is not only a crucial and secure source of vitamin A, but it can also be used to give extrudates a yellowish color [42]. The extrudates β-carotene level significantly changed after the inclusion of pumpkin flour. According to the ANOVA analysis, the blending ratio (lupin content) had no appreciable impact on the amount of β-carotene in linear terms (Table 5). However, the linear relationship between temperature of the barrel and the β-carotene content of extruded snack foods is substantial (p = 0.000). Adding 10–20 % more lupin flour did not change the amount of β-carotene in extrudates prepared with white rice flour. The concentration of β-carotene in cereal-based formulations has been shown to increase when pumpkin flour is added [80,79]. It is well known that adding high β-carotene ingredients can increase the amount of β-carotene in diets of cereal based foods [10]. Extrudates' β-carotene contents are decreased during extrusion processing. It can be related to the raw material's mechanical and heat degradation during extrusion processing [45,74].

The main pro-vitamin A component in the majority of carotenoid-containing foods is β-carotene, which is crucial to preventing vitamin A deficiency. The conversion of β-carotene to retinol equivalent (RE) is taken into account. 0.167 μg of RE equal 1 μg of β-carotene. For the pumpkin integrated extrudate, as indicated in Table 4, extrudates can supply pro-vitamin A in the range of 367–868 μg RE. However, the majority of the generated extrudates contain pro-vitamin A > 634 μg RE. In comparison to the control extrudate (zero microgram RE), pumpkin-incorporated extrudates had substantially greater amounts of β-carotene (RE). 600 mg of vitamin A per day as RE is required for teenagers between the ages of 10 and 18, according to the World Health Organization and Food and Agricultural Organization of the United Nations [WHO and FAO] [81]. Consequently, this project's extrudates may make a substantial contribution to RE, raising pro-vitamin A consumption and reducing the incidence of vitamin A deficiency.

### 3.4 Effect of extrusion processing parameters and mix ratios on sensory properties of an extruded snack made of white rice, lupin, and pumpkin

In addition to being important for product development, sensory evaluation also affects a product's acceptability and shelf life. A product does not have any economic importance if it is nutritionally superior but has lower acceptability in terms of taste and flavor [82]. Table 6 shows the impacts of blending ratio, feed moisture and barrel temperature level on customer acceptance of the snack foods' color, taste, flavor, texture, and overall acceptability as represented in their mean ratings. All extruded snack products from blends had sensory scores more than or equal to 5, which shows that the panelists enjoyed all of the products. White rice-lupin-pumpkin extruded snacks' sensory qualities were significantly impacted by the lupin and pumpkin flour content added. The extrudates' color acceptability ranged from 4.8 (control sample) to 6.5 (run 13). The addition of pumpkin and lupin flour improved the consumer appeal of the color of the extrudates. Pumpkin flour had yellowish colors added by organic yellow pigments, which the sensory panelists found more appealing than the control sample's pale color [19,83]. The degree of browning reactions, such as caramelization, the Maillard reaction, the degree of cooking, and pigment deterioration, are correlated with color changes in the extrudates. This happens throughout the extrusion process and is correlated with the formulation's composition on three different occasions [33,50,64]. The addition of pumpkin flour also had a positive linear effect on color intensity at higher extrusion temperatures, at which color scores of extrudates containing 4–8% pumpkin flour were concerned [42]. Similar findings were reported in studies conducted by other researchers when lupin flour was added up to 20 %, which had no discernible impact on the sensory score for color (consumer acceptance of color) in pasta and biscuits [64].

**Table 6:** Average ratings for various sensory characteristics of white rice-lupin-pumpkin extruded snack on 9 point hedonic scale.

**Run**	Independent variables			Sensory properties				
	**B**	**BT**	**FM**	Color	Taste	Flavor	Texture	Overall acceptability
1	15	135	17	5.9 ± 0.49^ABCD^	5.6 ± 0.65^ABCDE^	6.3 ± 0.48^AB^	6.5 ± 0.65^A^	6.4 ± 0.50^ABCD^
2	10	115	17	5.6 ± 0.50^D^	5.6 ± 0.50^ABCDE^	6.2 ± 0.69^AB^	6.2 ± 0.44^ABC^	6.3 ± 0.61^ABCD^
3	20	135	20	6.3 ± 0.63^ABC^	5.7 ± 0.63^ABCD^	6.1 ± 0.72^AB^	5.3 ± 0.48^HI^	6.0 ± 0.73^BCDEFG^
4	10	155	17	6.1 ± 0.28^ABCD^	5.8 ± 0.60^ABC^	6.5 ± 0.77^A^	6.2 ± 0.41^ABC^	6.2 ± 0.37^BCDEF^
5	15	115	20	6.2 ± 0.44^ABC^	5.2 ± 0.41^DE^	5.8 ± 0.37^BCD^	6.2 ± 0.37^ABC^	6.3 ± 0.49^ABCD^
6	20	115	17	6.0 ± 0.84^ABCD^	5.1 ± 0.28^E^	6.1 ± 0.83^AB^	5.2 ± 0.37^I^	5.6 ± 0.70^FG^
7	15	135	17	6.2 ± 0.62^ABC^	5.7 ± 0.69^ABCD^	6.0 ± 0.58^ABC^	6.1 ± 0.83^ABCDEF^	6.6 ± 0.51^AB^
8	15	115	14	6.1 ± 0.64^ABCD^	5.7 ± 0.56^ABCD^	5.4 ± 0.51^CD^	5.9 ± 0.28^BCDEFG^	5.6 ± 0.51^FG^
9	10	135	20	5.9 ± 0.78^BCD^	5.4 ± 0.50^BCDE^	6.1 ± 0.78^AB^	5.7 ± 0.48^DEFGHI^	5.6 ± 0.65^FG^
10	20	135	14	6.2 ± 0.69^ABC^	5.5 ± 0.65^ABCDE^	5.9 ± 0.67^BC^	5.9 ± 0.28^BCDEFG^	5.9 ± 0.81^CDEFG^
11	15	135	17	6.4 ± 0.70^AB^	5.6 ± 0.65^ABCDE^	6.1 ± 0.57^AB^	6.4 ± 0.71^AB^	6.5 ± 0.65^ABC^
12	15	155	14	6.2 ± 0.44^ABC^	5.8 ± 0.44^ABC^	6.3 ± 0.61^AB^	5.8 ± 0.37^CDEFGH^	6.8 ± 0.80^A^
13	20	155	17	6.5 ± 0.71^A^	5.4 ± 0.50^BCDE^	5.2 ± 0.60^D^	5.6 ± 0.77^GHI^	5.8 ± 0.55^DEFG^
14	10	135	14	5.8 ± 0.44^CD^	5.9 ± 0.28^AB^	6.1 ± 0.73^AB^	6.2 ± 0.71^ABC^	6.5 ± 0.59^ABC^
15	15	155	20	6.1 ± 0.73^ABCD^	5.3 ± 0.48^CDE^	5.9 ± 0.28^ABC^	5.6 ± 0.50^GHI^	5.5 ± 0.51^G^
	Control∗			4.8 ± 0.55^E^	6.0 ± 0.93^A^	5.8 ± 0.90^BCD^	5.6 ± 0.81^GHI^	5.7 ± 0.79^EFG^

Notes: ^A,B,C,D,E,F,H,I^ The results were presented as means ± standard deviation (n = 3); Means that differ by superscript within a column indicate a significant difference (p ≤ 0.05). B = Lupin Content (%); BT = Barrel Temperature (°C); FM = Feed Moisture (%).

The blended extrudates flavor acceptance ranged from 5.2 (run 13) to 6.5 (run 4). The product with 80 % white rice and 10 % lupin blend had the greatest flavor acceptance ratings, whereas the snack extrudate with 70 % white rice and 20 % lupin blend received the lowest ratings. In both linear and quadratic terms, the flavor score of extruded snack items is unaffected by the blending ratio (p = 0.684). However, the flavor of extrudates is significantly influenced by feed moisture content (p = 0.033). White rice, lupin, and pumpkin snacks' flavor acceptability was unaffected by the addition of 20 % lupin flour. Additionally, the panelists could not detect any beany flavor at low levels of lupin in the mix; this may be because higher cooking temperatures (115–155 °C) were important for changing and eradicating the off-flavors to enhance the sensory qualities of the ingredients [70].

The texture of the extrudate, which should have low hardness, is one aspect that influences whether or not the consumer will accept it. The texture and mouth feel of the white rice, lupin, and pumpkin extruded treats were significantly changed by the lupin and pumpkin integration. The extruded products' texture approval ranged from 5.2 (run 6) to 6.5 (run 1). However, with a few exceptions, all samples containing 10–20 % lupin and 10 % pumpkin flour were liked above 5 and were noticeably different. According to research, the extrudate's chewability, crispness, and hardness all affect how quickly it expands. Generally speaking, a product's expansion immediately affects its texture, making expanded products crispier [67]. Jayasena and Nasar-Abbas [19] observed comparable outcomes when Australian sweet lupin (*Lupinus angustifolius*) flour was used in bread, cookies, spaghetti, breakfast bars and muffins.

The presence of pumpkin and lupin affected up to 20 % of the sensory evaluation results for the flavor of the extruded snack. The linear and quadratic elements of the flavor score of extruded snack items are significantly influenced by the blending ratio (p = 0.025). Similar findings were noted; Jayasena and Nasar-Abbas [19] observed that the incorporation of 40–50 % lupin flour greatly reduced the final product's flavor and led to poorer rankings for taste.

The extruded snack's overall acceptance scores showed a significant influence of the inclusion of lupin flour up to 20 %. The overall acceptance results for extruded snacks in this investigation were in line with what Adem et al. [70] had found. While lupin was present in 15 % and 20 % of the samples, higher color scores were obtained, and overall acceptability somewhat increased as lupin concentration increased. In addition, when the amount of lupin in the blend is large, the beany flavor of the lupin flour may have a noticeable detrimental impact on the flavor and taste of the finished products. According to Jayasena and Nasar-Abbas [19], this tendency is valid. In this study, consumers generally accepted extruded snacks created from a combination of white rice, lupin, and pumpkin in practically all sensory qualities.

In comparison to the control sample, the samples made by adding 10–20 % lupin flour to extrudates were generally similarly loved by the panelists for taste, texture, and overall acceptability. The findings of earlier research that demonstrated that the acceptability of other food products did not decrease when lupin was included at specific levels are consistent with the findings of the present investigation. Bread's texture, flavor, acceptance, or appearance were all unaffected by the addition of lupin, which may substitute up to 10 % of the wheat flour [84]. The 20 % level of lupin flour addition had no effect on the overall acceptability of instant noodles, but it significantly raised on color preference [19]. However, the addition of 40 % and 50 % lupin flour significantly decreased the final product's acceptability overall. The outcomes are consistent with those of Hall and Johnson [84], who found that samples containing lupin flour scored less favorably overall than control pasta samples. Additionally, the 10 % substitution of pumpkin flour in sweet bread cookies and sandwich bread did not substantially differ from the control (p > 0.05), with acceptability scores ranging from “like slightly” to “like moderately” [56].

### 3.5 Optimal conditions for the highest quality extruded white rice, lupin, and pumpkin snack product

**Fig. 1:**
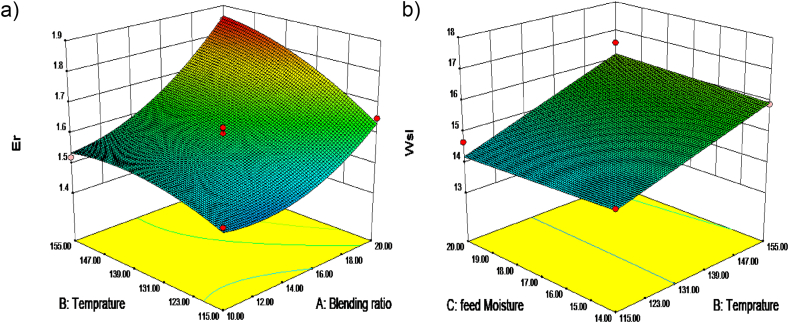
Response surface plot on independent variables. a) ER and b) WSI.

**Fig. 2:**
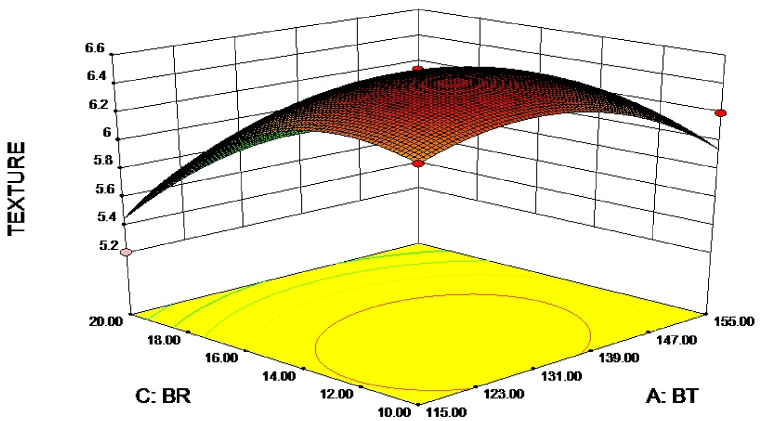
Response surface plot of texture on independent variables.

The RSM was used to improve the formula in order to create a consumer-friendly, highly nutritious extruded snack. The correlations between the independent factors (blending ratio, feed moisture content and barrel temperature) and the dependent variables were investigated using numerical optimization in Design Expert ver. 7. According to Bas and Boyaci [85], the physical, functional, proximate composition, micronutrients, and sensory qualities were all examined properties that were thought to have a significant impact on the product's quality. Higher ER, WSI, protein, fiber, iron, zinc, β-carotene, and texture characteristics were targeted through process parameter optimization. For the graphical optimization of the overlay plot, the projected values of ER (1.9), WSI (17.8), protein% (13.8), and texture 6.4 were employed (see Fig. 1, Fig. 2). The optimal extrusion parameters were a 15 % lupin mix, 155 °C barrel temperature, and 14 % feed moisture, which resulted in a snack meal with a desire value of 0.75.

## 4 Conclusion

The proximate composition and micro-nutrient content of the extrudates were significantly influenced (p < 0.05) by the extrusion circumstances, as well as physical and functional features including BD, ER, WSI, and WAI. Extruded snack food with low feed moisture content and mixing ratios expanded effectively and had lower BD. The WSI decreased and the WAI increased in proportion to the rise in feed moisture. As the amount of lupin increased, the values of the ER, WAI, and WSI dropped. The extrudates from the mixture consisting of white rice, lupin, and pumpkin flour were more nutritious. The study finds that lupin flour, which has high levels of protein, fat, and dietary fiber, can be successfully employed in a variety of food products up to a particular amount without diminishing the acceptance of those products among consumers. White rice-lupin-pumpkin extruded snack meals had higher lupin additions, which was reflected in the rises in protein, crude fiber, ash, fat, iron, and zinc contents. Moreover, the addition of pumpkin flour greatly raised the β-carotene content of the extruded products. The results showed that the amounts of lupin substitution and the addition of pumpkin flour had significantly changed the extruded products' protein, crude fiber, ash, β-carotene, iron, and zinc contents. Compared to the control 100 % white rice extruded snacks, the extruded snacks with the addition of lupin and pumpkin flour had higher protein, ash, fiber, fat, β-carotene, iron, and zinc contents while having significantly lower total carbohydrate contents. Overall sensory acceptability findings suggested that up to a specific amount of lupin could be added to foods without negatively influencing their consumer appeal. 15 % blending ratio, 155 °C barrel temperature, and 14 % feed moisture were found through optimization. The final snack food product would have a 1.6 % ER, 15.9 % WSI, 17.3 % protein content, and an overall acceptability of 6.8 (on a 9-point hedonic scale). Current findings may be used to produce extruded products with an enhanced fiber content, higher beta-carotene, protein, iron, and zinc content, and an increased quality within white rice-lupin-pumpkin based products, even though the exact interaction needs further study to be determined.

## Ethical approval and consent to participate

This study was approved by Institutional Review Board of Bahir Dar University. There are no human subjects in this article, and an informed consent is not applicable. Verbal consent was obtained from the panelists regarding their voluntary participation in the sensory evaluation of the product. It is not possible or unusual to obtain written approval from the country's legal organization for sensory experiments.

## CRediT authorship contribution statement

**Yeshambel Dagnaw Alefew:** Writing – original draft, Visualization, Supervision, Project administration, Investigation, Formal analysis, Data curation, Conceptualization. **Abebaw Teshome Tiruneh:** Writing – review & editing, Visualization, Supervision, Methodology, Investigation, Formal analysis, Data curation. **Tadesse Fenta Yehuala:** Writing – review & editing, Validation, Supervision, Methodology.

## Data availability statement

The datasets during and/or analysed data used to support the findings of the present study are available from the corresponding author upon request.

## Funding statement

The research conducted in this study is supported by funding from Bahir Dar Institute of Technology, School of Research and Graduate Studies, 10.13039/501100005872Bahir Dar University, Ethiopia.

## Declaration of competing interest

The authors declare that they have no known competing financial interests or personal relationships that could have appeared to influence the work reported in this paper.

## References

[1] Filli K.B. (2016). Physicochemical properties of sorghum malt and Bambara groundnut based extrudates. J. Food Sci. Technol. Nepal.

[2] Patel D., Rathod R. (2017). Ready to eat food perception, food preferences and food choice: a theoretical discussion. World Wide J. Multidisciplin. Res. Dev.

[3] Brennan M.A. (2012). Enrichment of extruded snack products with coproducts from chestnut mushroom (*Agrocybe aegerita*) production: interactions between dietary fiber, physicochemical characteristics, and glycemic load. J. Agric. Food Chem..

[4] Mahanani R.S. (2023). Physical characteristics of extrudate from mixed corn gritsoybean flour with treatments of moisture content and extruder barrel temperature. BIO Web Confer..

[5] Promsakha na Sakon Nakhon P. (2018). Optimization of pumpkin and feed moisture content to produce healthy pumpkin-germinated brown rice extruded snacks. Agri. Nat. Res..

[6] Leonard W. (2020). Application of extrusion technology in plant food processing byproducts: an overview. Compr. Rev. Food Sci. Food Saf..

[7] Pathak N., Kochhar A. (2018). Extrusion technology: solution to develop quality snacks for malnourished generation. Int. J. Current Microbiol. Appl. Sci..

[8] Anton A.A., Luciano F.B. (2007). Instrumental texture evaluation of extruded snack foods: a review. Cienciay Tecno-logia Alimentaria.

[9] Alonso R., Orue E., Marzo F. (1998). Effects of extrusion and conventional processing methods on protein and antinutritional factor contents in pea seeds. Food Chem..

[10] Delgado-Nieblas C.I. (2015). Elaboration of functional snack foods using raw materials rich in carotenoids and dietary fiber: effects of extrusion processing. CyTA - J. Food.

[11] Mbatchou V.C., Dawda S. (2013). The nutritional composition of four rice varieties grown and used in different food preparations in Kassena-Nankana District, Ghana. Int. J. Res. Chem. Environ.

[12] Bhat T. (2020). Development of thiamine-rich snacks from brown rice using extrusion technology. Br. Food J..

[13] Verma D.K., Srivastav P.P. (2017). Proximate composition, mineral content and fatty acids analyses of Aromatic and non-Aromatic Indian rice. Rice Sci..

[14] Kumar N., Sarkar B.C., Sharma H.K. (2010). Development and characterization of extruded product using carrot pomace and rice flour. Int. J. Food Eng..

[15] Awolu O.O., Osigwe M.A. (2019). Nutritional and antioxidant potential of rice flour enriched with Kersting's groundnut (*Kerstingiella geocarpa*) and lemon pomace. Int. J. Food Stud..

[16] Ertaş N., Bilgiçli N. (2014). Effect of different debittering processes on mineral and phytic acid content of lupin (*Lupinus albus L.*) seeds. J. Food Sci. Technol..

[17] Zerihun N. (2012). Contribution of white lupin (*Lupinus albus L.*) for food security in north-western Ethiopia: a review. Asian J. Plant Sci..

[18] Carvajal Larenas F.E., Koziol M.J., Caviedes M. (2024). Could snacks based on lupin Be a nutritious treat? A point of view. Foods.

[19] Jayasena V., Leung P.P.Y., Nasar-Abbas S.M. (2010). Effect of lupin flour substitution on the quality and sensory acceptability of instant noodles. J. Food Qual..

[20] Azeze H. (2016). Challenges on production and utilization of white lupin (*Lupinus albus L*.) in Ethiopia: a strategic orphan crop. Am. J. Exp. Agric..

[21] Zelalem K.A., Chandravanshi B.S. (2015). Levels of essential and non-essential elements in raw and processed. African Soc. Sci. J..

[22] Yeheyis L. (2011). Effect of a traditional processing method on the chemical composition of local white Lupin (*Lupinus albus L.*) seed in north-western Ethiopia. Zeitschrift fur Naturforschung - Sec. C J. Biosci..

[23] Tharanathan R.N., Mahadevamma S. (2003). Grain legumes a boon to human nutrition. Trends Food Sci. Technol..

[24] Promsakha na Sakon Nakhon P. (2017). Comparisons of physicochemical properties and antioxidant activities among pumpkin (*Cucurbita moschata L.*) flour and isolated starches from fresh pumpkin or flour. Int. J. Food Sci. Technol..

[25] Staichok A.C.B. (2016). Pumpkin peel flour (*Cucurbita máxima L*.)-Characterization and technological applicability. J. Food Nutr. Res..

[26] Ghendov-Mosanu A. (2023). Effect of bioactive compounds from pumpkin powder on the quality and textural properties of shortbread cookies. Foods.

[27] Murzaini N.M.N. (2020). Effect of pre-treatment in producing pumpkin powder using air fryer and its application in 'bingka' baking. Curr. Res. Nutr Food Sci Jour..

[28] UNDP (2006).

[29] Nugent R. (2020). Economic effects of the double burden of malnutrition. Lancet.

[30] Wakeel A. (2018).

[31] Mabhaudhi T. (2016). Opportunities for underutilised crops in Southern Africa's post-2015 development agenda. Sustainability.

[32] Chivenge P. (2015). The potential role of neglected and underutilised crop species as future crops under water scarce conditions in Sub-Saharan Africa. Int. J. Environ. Res. Publ. Health.

[33] Sharmila B., Athmaselvi K.A. (2017). Development of ready to eat extruded snacks from blend of under-utilized legumes and millets. J. Pharmaceut. Sci. Res..

[34] Szpisják-Gulyás N. (2023). Methods for experimental design, central composite design and the Box–Behnken design, to optimise operational parameters: a review. Acta Aliment..

[35] Liu Y. (2021). Optimisation of the extrusion process through a response surface methodology for improvement of the physical properties and nutritional components of whole black-grained wheat flour. Foods.

[36] Getachew P. (2012). Proximate composition and anti-nutritional factors of traditionally processed white lupine (*Lupinous albus L.*) fabaceae, grown in Ethiopia. Ethiop. J. Biol. Sci..

[37] Arachchige U.S.P.R. (2019). Development of extruded snacks using pumpkin flour. Int. J Sci. Technol. Res..

[38] Khan M.A. (2019). Effect of pumpkin flour on the rheological characteristics of wheat flour and on biscuit quality. J. Food Process. Technol..

[39] Saleh E.H., Mansour E.H., Youssef H.E. (2019). 6^th^ Annual International Scientific Conference, Specific Studies and Their Role in Activating Tourism for the Development of the National Economy.

[40] Lazic Z.R. (2004).

[41] Myers R., Montgomery D., Anderson-Cook C. (2016).

[42] Obradovic (2015).

[43] Fan J., Mitchell J.R., Blanshard J.M.V. (1996).

[44] Ibanoglu S., Ainsworth P., Özer E.A., Plunkett A. (2006). Physical and sensory evaluation of a nutritionally balanced gluten-free extruded snack.

[45] Stojceska V. (2008). Cauliflower by-products as a new source of dietary fibre , antioxidants and proteins in cereal based ready-to-eat expanded snacks.

[46] Anderson R.A., Conway H.F., Peplinski A.P.I. (1970). Gelatinization of corn grits by roll cooking. Extrus. Cook. Steam..

[47] Tiruneh A.T. (2020). Effect of mango and carrot fortification on proximate composition, β-carotene and sensory properties of teff Injera. Cogent Food Agric..

[48] Monro J., Burlingame B. (1996). Carbohydrates and related food components: INFOODS tagnames, meanings, and uses. J. Food Compos. Anal..

[49] Arribas C. (2019). Extrusion effect on proximate composition, starch and dietary fibre of ready-to-eat products based on rice fortified with carob fruit and bean. Lwt. Elsevier.

[50] Algarni E.H., Hussien H.A., Eman M.S. (2019). Development of nutritious extruded snacks. Life Sci. J..

[51] Salehi F. (2016). Potential of sponge cake making using infrared-hot air dried carrot. J. Texture Stud..

[52] Deshpande H.W., Poshadri A. (2011). Physical and sensory characteristics of extruded snacks prepared from Foxtail millet based composite flours. Int. Food Res. J..

[53] Awolu O.O., Magoh A.O., Ojewumi M.E. (2020). Development and evaluation of extruded ready-to-eat snack from optimized rice, kersting's groundnut and lemon pomace composite flours. J. Food Sci. Technol. Springer India.

[54] Dushkova M. (2023). Physicochemical and sensory characteristics of extrudates from rice enriched with pumpkin. Food Sci. Appl. Biotechnol..

[55] Philipp C. (2017). Instrumental and sensory properties of pea protein-fortified extruded rice snacks. Food Res. Int..

[56] Pongjanta J. (2006). Utilization of pumpkin powder in bakery products. Songklanakarin J. Sci. Technol..

[57] Dhiman A.K. (2018). Preparation of pumpkin powder and pumpkin seed kernel powder for supplementation in weaning mix and cookies. Int. J. Chem. Stud..

[58] Khatib El S., Muhieddine M. (2020). The Health Benefits of Foods - Current Knowledge and Further Development.

[59] Kefale B., Abrha E. (2018). Sweet lupine recipe development and nutritional content of recipe at Holeta, Ethiopia. J. Food Sci. Nutr. Therapy.

[60] Jahreis G. (2016). Legume flours: nutritionally important sources of protein and dietary fiber. Ernahrungs Umsch..

[61] Ka Z., Chandravanshi B.S. (2014). Levels of essential and non-essential elements in raw and processed Lupinus albus (*white lupin, Gibto*) cultivated in Ethiopia. Afr. J. Food Nutr. Sci..

[62] Swamy B.P.M. (2019). Compositional analysis of genetically engineered GR2E “golden rice” in comparison to that of conventional rice. J. Agric. Food Chem..

[63] Wang S., Errington S., Yap H.H. (2008). Proceedings of the 12th International Lupin Conference.

[64] Oliveira C.T. (2015). Development and characterization of extruded broken rice and lupine (lupinus albus). Am. J. Plant Sci..

[65] Devi N.L. (2013). Development of protein-rich sorghum-based expanded snacks using extrusion technology. Int. J. Food Prop..

[66] Pramesh K.D., Arti C., Sukhcharn S. (2014). Evaluation of extrudate from sweetpotato flour and tomato pomace blend by extrusion processing. Afr. J. Food Sci..

[67] Kaisangsri N. (2016). Carrot pomace enhances the expansion and nutritional quality of corn starch extrudates. LWT - Food Sci. Technol. (Lebensmittel-Wissenschaft -Technol.).

[68] Hagenimana A., Ding X., Fang T. (2006). Evaluation of rice flour modified by extrusion cooking. J. Cereal. Sci..

[69] Rathod R.P., Annapure U.S. (2017). Physicochemical properties, protein and starch digestibility of lentil based noodle prepared by using extrusion processing. LWT--Food Sci. Technol..

[70] Adem M. (2019). Optimization of lupine (*Lupinus albus L.*) composition, feed moisture content and barrel temperatures for best quality maize based extruded snack food. Nutr. Food Sci..

[71] Singh S., Gamlath S., Wakeling L. (2007). Nutritional aspects of food extrusion: a review. Int. J. Food Sci. Technol..

[72] Pardhi S.D. (2019). Evaluation of functional properties of extruded snacks developed from brown rice grits by using response surface methodology. J. Saudi Soc. Agri. Sci..

[73] Pansawat N. (2008). Effects of extrusion conditions on secondary extrusion variables and physical properties of fish, rice-based snacks. LWT - Food Sci. Technol. (Lebensmittel-Wissenschaft -Technol.).

[74] Singha P., Singh S.K., Muthukumarappan K., Krishnan P. (2018). Physicochemical and nutritional properties of extrudates from food grade distiller's dried grains, garbanzo flour, and corn grits. Food Sci. Nutr..

[75] Coutinho L.S. (2013). Optimization of extrusion variables for the production of snacks from by-products of rice and soybean. Food Sci. Technol..

[76] Okunola A.A. (2023). Development and process optimization of a ready-to-eat snack from rice-cowpea composite by a twin extruder. Processes.

[77] Ziena H.M., Ziena A.H.M. (2022). Nutritious novel snacks from some of cereals, legumes and skimmed milk powder. Appl. Food Res..

[78] Bassinello P.Z. (2015). Expanded gluten‐free extrudates made from rice grits and bandinha (bean) flour mixes: main quality properties. J. Food Process. Preserv..

[79] Kundu H., Grewal R.B., Goyal A., Upadhyay N., Prakash S. (2012). Effect of incorporation of pumpkin (Cucurbita moshchata) powder and guar gum on the rheological properties of wheat flour. J. Food Sci. Technol..

[80] Sathiya Mala K. (2018). Effect of pumpkin powder incorporation on the physico-chemical, sensory and nutritional characteristics of wheat flour muffins. Int. Food Res. J..

[81] World Health Organization and Food and Agricultural Organization of the United Nations [WHO and FAO] (2004). Report of a Joint FAO/WHO Expert Consultation.

[82] Yadav U., Singh R.R.B., Arora S. (2018). Evaluation of quality changes in nutritionally enriched extruded snacks dur-ing storage. J. Food Sci. Technol..

[83] Turksoy S., Özkaya B. (2011). Pumpkin and carrot pomace powders as a source of dietary fiber and their effects on the mixing properties of wheat flour dough and cookie quality. Food Sci. Technol. Res..

[84] Hall R.S., Thomas S.J., Johnson S.K. (2005). Australian sweet lupin flour addition reduces the glycaemic index of a white bread breakfast without affecting palatability in healthy human volunteers. Asia Pac. J. Clin. Nutr..

[85] Bas I., Boyaci I.S. (2007). Modelling and optimization I: usability of response surface methodology. J. Foodserv..

